# Variation at Innate Immunity Toll-Like Receptor Genes in a Bottlenecked Population of a New Zealand Robin

**DOI:** 10.1371/journal.pone.0045011

**Published:** 2012-09-14

**Authors:** Catherine E. Grueber, Graham P. Wallis, Tania M. King, Ian G. Jamieson

**Affiliations:** 1 Department of Zoology, University of Otago, Dunedin, New Zealand; 2 Allan Wilson Centre for Molecular Ecology and Evolution, Palmerston North, New Zealand; University of Uppsala, Sweden

## Abstract

Toll-like receptors (TLRs) are an ancient family of genes encoding transmembrane proteins that bind pathogen-specific molecules and initiate both innate and adaptive aspects of the immune response. Our goal was to determine whether these genes show sufficient genetic diversity in a bottlenecked population to be a useful addition or alternative to the more commonly employed major histocompatibility complex (MHC) genotyping in a conservation genetics context. We amplified all known avian TLR genes in a severely bottlenecked population of New Zealand's Stewart Island robin (*Petroica australis rakiura*), for which reduced microsatellite diversity was previously observed. We genotyped 17–24 birds from a reintroduced island population (including the 12 founders) for nine genes, seven of which were polymorphic. We observed a total of 24 single-nucleotide polymorphisms overall, 15 of which were non-synonymous, representing up to five amino-acid variants at a locus. One locus (*TLR1LB*) showed evidence of past directional selection. Results also confirmed a passerine duplication of *TLR7*. The levels of TLR diversity that we observe are sufficient to justify their further use in addressing conservation genetic questions, even in bottlenecked populations.

## Introduction

The evolutionary consequences of the amount and distribution of genetic diversity among individuals are a major focus of population and conservation genetics. Widely-used markers such as microsatellites are useful for estimating migration and delineating management units, but are not necessarily relevant to processes affecting evolutionarily significant functional diversity [1]. An understanding of patterns of functional genetic diversity is helpful to elucidate causes of individual-level variation in fitness generally or, in a conservation context, to examine processes that may influence population-level adaptive potential. Studies of functional diversity in non-model vertebrate species have been dominated by examination of the major histocompatibility complex (MHC), a group of genes encoding cell-surface proteins responsible for binding and presentation of foreign peptides [2]. MHC genes exhibit extremely high levels of genetic and genomic (copy-number) diversity, both within and among taxa. They are highly informative with respect to individual and perhaps population viability due to their direct association with immune function [3], [4], [5]. However, these high levels of diversity, particularly resulting from gene duplications, can present a challenge to the study of MHC loci in non-model organisms because coamplifying loci interfere with haplotype phasing and genetic diversity estimates [6]. Furthermore, in humans (and potentially other vertebrates), at least half of the genetic basis of inter-individual variability in immune responses to pathogens is thought to be a consequence of non-MHC genes [7], [8]. It is becoming increasingly recognised that a broader approach to the study of wildlife functional genetic diversity, beyond MHC, is required [8], [9], [10], [11].

Toll-like receptor (TLR) genes are an ancient part of the genome of many animals. They mediate innate immune responses via recognition of pathogen-associated molecular patterns (PAMPs) such as dsRNA of some viruses, or lipopolysaccharide of Gram-negative bacteria [12], [13], [14]. As a result of PAMP binding, TLRs initiate a cascade of intracellular signalling responses, stimulating many genes associated with inflammation and immunity, including those of the acquired immune response (such as MHC pathways) [12], [15], [16], [17]. The genomes of many animal lineages contain a suite of TLR genes that facilitate recognition of a variety of different PAMPs. For example, mediation of immune response to Gram-negative bacteria (e.g. *Escherichia coli*) involves *TLR4* [18], [19]. Response to Gram-positive bacteria (e.g. *Staphylococcus aureus*) has been shown in mammals to be linked to both *TLR1* [20] and *TLR2* [19], genes that are both duplicated in birds (named *TLR1LA* and *TLR1LB*, and *TLR2A* and *TLR2B* [21]). Response to eukaryote fungi has been shown to be linked to *TLR2* in mammals [22]. Ligand sharing by multiple TLRs (i.e. multiple TLRs targeting similar PAMPs), as well as heterodimerisation between genes (such as formation of complexes between *TLR2* and *TLR1* or *TLR6*) have both been reported [20]. In birds, about ten TLRs is the norm [23], with one locus (*TLR15*) specific to birds and thought to be involved in response to other Gram-negative bacteria including *Salmonella enterica* [24], [25].

Growing evidence from studies of model organisms has indicated that variation in binding regions of TLR loci can translate to functional variation in pathogen recognition [26], [27], [28], [29]. Such variation is a likely contributor to the host-pathogen arms-race, and maintenance of high levels of population-wide variation at TLR loci may reflect the potential of populations to adapt to changing environments on evolutionary timescales [8], [28], [30], [31]. As such, positive and/or balancing selection have both been reported to drive the evolution of some mammalian TLR genes (e.g. [32], [33], [34], [35]), as well as some avian TLRs (e.g. [23], [31]).

Due to their key role in host defence, observed variation in TLR genes likely represents an important component of functional genetic diversity of wild populations [8], [9], [23]. When general estimates of putatively functional genetic diversity are of interest, examining TLR gene diversity may be a promising alternative (or addition) to MHC. This is particularly true for passerines, in which the MHC is especially complex, causing genotype reconstruction to be difficult and/or costly [36], [37], [38], [39]. The reduced genomic complexity of most TLR genes may help overcome many of these issues, allowing diversity estimates for multiple genes, recognising a variety of PAMPs, to be obtained relatively easily.

Recent investigations of TLR diversity have focussed on model and abundant species [10], [23], [28], [31], [40], but whether these genes are variable in bottlenecked populations, where genetic variation is usually greatly reduced, has not been explored. Here we report variation at the majority of TLR loci in a bottlenecked population of an endemic robin (*Petroica australis rakiura* [41]) found on Stewart Island (174,600 ha), New Zealand's third largest island. Previously common and widespread, *P. a. rakiura* declined to <300 individuals in approximately 1% of its former habitat since the introduction of mammalian predators to Stewart Island over the last four centuries [42]. The population is therefore expected to have reduced genetic diversity as a result of this demographic bottleneck. A further bottleneck event occurred when, in 2000/2001, 25 individuals from a remnant population on Stewart Island were used to reintroduce robins to nearby Ulva Island (257 ha) from which introduced predators had been eradicated. Twelve of these individuals (five females and seven males) successfully contributed to the descendent population inhabiting the island today [43]. Previous studies using microsatellite genotyping of Stewart Island robins have supported the prediction of reduced genetic diversity as a result of population bottlenecking [44], [45], [46]. In the current study, we quantify the levels of TLR genetic diversity present in *P. a. rakiura* by specifically examining diversity in the 12 Ulva Island founders [43]. Diversity observed among these birds provides an indication of the levels of diversity present in the mainland population, as well as an estimate of the maximum level of diversity likely to be present among the current, reintroduced Ulva Island population. To our knowledge, this study is the first multigene assay of TLR genetic diversity in a threatened species.

## Materials and Methods

### Samples

DNA samples had previously been collected from ten of the Ulva Island genetic founders (one male and one female founder died prior to sample collection). To deduce the missing founders' genotypes, we included up to seven offspring from each, as well as the other parents of those offspring. For loci where the sampled parent or offspring were homozygous, we could infer the genotypes of the unsampled parents based on a smaller number of offspring genotypes (see Results). For other loci, we genotyped a minimum of five offspring, ensuring <3.5% probability of failing to observe an unknown parent's allele (where *p* = 1/2*^N^* ^.offspring^, following [47]). DNA samples were prepared for a previous study [45] and stored at −20°C until use.

### TLR primer optimisation and development

Alcaide and Edwards [23] developed primers targeting the extracellular domain of all known avian TLRs, which they tested in a number of avian families. In *P. a. rakiura*, we were able to use primers from their main set (Table S1 in [23]) to amplify five genes: *TLR1LA*, *TLR1LB*, *TLR3*, *TLR5* and *TLR15*, while *TLR7* amplified more reliably with the alternative set (Table S1 in [23]). *TLR4* did not amplify consistently with *avTLR4* [23], so we designed species-specific internal primers using Primer3 [48]: *PauTLR4F* 5′ GCTTTCCTTGAACAACATAAAGTCC 3′ and *PauTLR4R* 5′ GGGACAGAAAGACAGGGTAGG 3′ that produce a 776-bp product. Amplification of *TLR21* with *avTLR21* or *avTLR21-2* [23] failed to produce a clear band on agarose so we designed primers based on passerine *TLR21* sequences available on Genbank (house finch *Carpodacus mexicanus*: GU904987 and zebra finch *Taeniopygia guttata*: NW_002218839) – *finchTLR21F* 5′ TTGACAACAACCTGCTCACTG 3′ and *finchTLR21R* 5′ TACGCAGCTCGTTCTTGG 3′ (831-bp product). Our *TLR21* primers improve identity with passerines, as ambiguities in the multi-species avian alignment [23] are assigned the conserved nucleotides observed in the two finch species.

Sequence data for *TLR2* using previously published primers [23] showed coamplification of the two genes *TLR2A* and *TLR2B* resulting in numerous polymorphic sites. In order to design locus-specific primers to allow us to amplify the two genes separately, a coamplified *TLR2A* and *TLR2B* PCR product obtained using primers *avTLR2F* & *R* [23] was cloned using the pGEM-T Easy vector (Promega) following the manufacturer's instructions, and transformed into TOP 10 (Invitrogen) competent cells. We obtained sequence data for each gene (*TLR2A* and *TLR2B*) by sequencing seven clones from one individual and comparing the results to published sequences on Genbank using a BLAST search [49]. Locus-specific forward primers (*PauTLR2AF* 5′ CCAAAGTATTACCCGAGCAATAGTG 3′, and *PauTLR2BF* 5′ CCTAAGATCGCCAGCATTTTAG 3′) were designed in a region of high dissimilarity between the two genes (16% and 18% respective similarity to the other gene). These forward primers were each paired with *avTLR2R* [23] to produce 1,147-bp and 1,137-bp products respectively, and used to amplify the two genes separately for all founders.

Sequence data for *TLR7* also suggested coamplification of duplicate loci by the presence of eight nucleotide sites (0.8% of 947 bp) that appeared heterozygous for all genotyped individuals (with uniform chromatogram peak heights). These SNPs also did not appear to follow Mendelian inheritance, as this dataset included 15 parent/offspring trios in which all individuals were heterozygous (under Mendelian inheritance we expect 50% of the offspring of two heterozygous parents to be heterozygotes; the probability of observing 15/15 heterozygous offspring is 3×10^−5^), further indicating coamplification of duplicate loci. Three additional sites at *TLR7* showed variable heterozygosity (variation in peak heights among individuals) among sampled birds suggesting that one or both genomic copies of this gene may be variable. Due to the close similarity between duplicate copies of *TLR7* in *P. a. rakiura*, locus-specific primers could not be designed. However, as a preliminary investigation into the structure and diversity of this gene we cloned PCR products from two unrelated individuals amplified using *avTLR7-2 F* & *R* [23]. A PCR product was amplified in three reactions, each using a different high-fidelity (proof-reading) enzyme: AccuSure and Velocity (both from Bioline Ltd) and Phusion (New England Biolabs). If there are two gene-copies, a maximum of four haplotypes per individual is expected. For the AccuSure enzyme, we used the following variations from standard PCR conditions (below), as recommended by the manufacturer: MgCl_2_ concentration of 2.0 mM (included in the enzyme buffer), initial hot-start at 95°C for 10 min and extension time of 2 min at 68°C. The Velocity and Phusion reagent setups followed the manufacturer's protocols, and thermocycling conditions did not vary from our standard protocol below, with the exception of a 98°C denaturation temperature. A total of 24–33 clones was sequenced for each individual. Sequencing 24 clones for an individual gives a 99.6% probability of observing all haplotypes at least once, if there are two heterozygous gene-copies (i.e. maximum of four unique haplotypes), based on multinomial probability [50].

### PCR conditions and sequencing

Unless described otherwise, all amplifications were carried out in a total volume of 15 µl and contained 0.6 U BioTaq DNA polymerase (Bioline Ltd), 200 µM each dNTP, 1.5 mM MgCl_2_, 1× Taq buffer and 500 nM each primer (Sigma-Aldrich). Thermocyling conditions on an Eppendorf Mastercycler pro S consisted of 94°C for 3 min, followed by 35 cycles of 94°C for 40 s, locus-specific annealing temperature ([23]; *PauTLR2A* T_a_ = 55°C; *PauTLR2B* T_a_ = 55°C; *PauTLR4* T_a_ = 60°C; *finchTLR21* T_a_ = 57°C) for 40 s, and extension at 72°C for 80 s, with a final extension step of 72°C for 10 min. Amplified products were purified by excision of the target band from a 1% agarose 1× TAE gel stained with SYBR Safe DNA gel stain (Invitrogen) and cleaned up using the DNeasy Ultra-Sep gel purification kit (Omega Bio-Tek). Purified PCR products were sequenced in both directions on an ABI 3730xl Genetic Analyser (Genetic Analysis Services at Otago). Correct amplification of the target genes was confirmed by BLAST search.

Sequences were edited and aligned using Sequencher v5.0 (Gene Codes Corporation), using IUPAC ambiguity codes where double-peaks were observed. All SNPs for all individuals were confirmed by careful examination of chromatograms; any putative SNPs or haplotypes observed in only one individual were re-amplified and sequenced. None of the individuals included in the analyses (and reported in the results) had any missing data for any SNPs. Sequence data from this manuscript is available on Genbank (accession numbers provided in Table 1).

**Table 1:** Polymorphism of the exons encoding the pathogen-recognition extracellular domains of Toll-like receptor genes among Stewart Island robin *Petroica australis rakiura* founders of Ulva Island.

Gene (target exon)	*N* 1	Fragment length (bp)	Observed haplotypes	SNPs	Amino acids	syn∶nsyn	π (±SE)^2^	Genbank accession
*TLR1LA* (ex 2)	10 founders (+2 & 5)	1,166	2	172: C/T	Arg/Trp	1∶1	8.89 (6.84)	JX502625: JX502627
				291: G/A	Ser			
*TLR1LB* (ex 1)	10 founders (+2 & 5)	971	2	154: C/T	Leu	2∶1	16.01 (11.04)	JX502628: JX502630
				185: C/T	Ala/Val			
				369: G/A	Val			
*TLR2A* (ex 2)	10 founders (+6 & 5)	1,034	2	891: C/T	Gly	1∶0	5.01 (4.86)	JX502631: JX502633
*TLR2B* (ex 2)	10 founders (+7 & 6)	1,021	3	108: G/A	Glu	4∶1	21.40 (13.68)	JX502634: JX502637
				324: C/T	Asn			
				632: C/T	Thr/Met			
				645: C/T	Asn			
				846: G/A	Arg			
*TLR3* (ex 4)	9 founders (+5^3^ & 5)	1,087	1	(none)	-	-	-	JX502638
*TLR4* (ex 3)	10 founders (+5 & 6)	649	5	94: A/C/T	Ile/Leu/Phe	0∶4	27.13 (18.17)	JX502639: JX502644
				142: C/T	Leu/Phe			
				220: A/T	Thr/Ser			
				520: G/A	Asp/Asn			
*TLR5* (ex 1)	10 founders (+2 & 5)	1,229	3	371: G/A	Arg/Gln	0∶2	4.66 (4.36)	JX502645: JX502648
				817: G/T	Asp/Tyr			
*TLR7* (ex 2)^4^	10 founders (+6 & 7)	1,010^5^	≥2	162: G/A	Asn/Asp	0∶3	N/A^4^	JX502657: JX502665
				672: C/T	Pro/Ser			
				723: G/A	Ser/Gly			
*TLR15* (ex 1)	10 founders (+6 & 6)	1,279	2	997: G/A	Asp/Asn	0∶1	0.65 (1.40)	JX502649: JX502651
*TLR21* (ex 1)	10 founders (+7 & 7)	618	4	118: G/A	Asn/Asp	1∶2	14.42 (11.62)	JX502652: JX502656
				169: C/T	Arg/Trp			
				300: G/A	Pro			
Mean		1,006	≥2.6	2.4		9∶15		

1 Numbers in brackets are the number of offspring used to infer the genotypes of the missing 11^th^ (female) and 12^th^ (male) founders. See Methods.

2 Nucleotide diversity (×10^4^).

3 These five birds are also the offspring of the 10^th^ founder that could not be genotyped for this locus.

4 *TLR7* primers co-amplified two loci, excludes SNPs that clearly do not follow Mendelian inheritance (likely to be fixed differences). Genotypes could not be inferred.

5 Length of cloned product (see Results); initial, population-level sequence analyses were based on directly sequenced PCR products (sequence length = 947 bp).

### Genetic diversity

Allelic phasing of SNPs was undertaken using a combination of PHASE v2.1.1 [51], [52] using default settings as implemented in DNAsp v5.1 [53], and comparison to known parent-offspring relationships. To avoid problems with non-independence of large numbers of parent-offspring samples in the dataset, analyses were only performed on the genotypes of the founder individuals, after the missing founder genotypes were inferred (i.e. *N* = 12 birds). Observed heterozygosity (*H*_O_) and expected heterozygosity (*H*_E_) as well as nucleotide diversity (π), were calculated using Arlequin 3.5 [54].

### Tests of selection

The McDonald-Kreitman statistic [55], implemented in DNAsp, was used to test for signatures of positive selection over long timescales by comparing the ratios of synonymous and non-synonymous substitutions that are fixed and polymorphic across divergent species [55], [56]. This test requires a second species from which multiple individuals are sampled, where the degree of relatedness to the focal species determines the timescale over which selection is determined [57]. We used sequences from *C. mexicanus* published by Alcaide and Edwards [23] because the sample sizes they used were generally similar to ours (*N* = 51 for *TLR1LA*, 13 for *TLR1LB*, 4 for *TLR21*, and 8 for *TLR2A*, *TLR2B*, *TLR4* and *TLR15* [23]). We did not perform the test for *TLR5*, as Alcaide and Edwards [23] report a possible pseudogene in *C. mexicanus* for this locus. Sequences were aligned using ClustalW codon alignment [58] implemented in MEGA5 [59]. Although our population is known to have undergone a demographic bottleneck, this should affect all genes equally and differences among genes may still be informative of selective processes [10], [57], [60].

## Results

### Levels of polymorphism

We successfully generated an average of 1,006 bp of sequence per gene for the full known complement of avian Toll-like receptors in *P. a. rakiura* (Table 1). Individual alleles, based on SNP haplotypes, could be easily assigned for nine loci from Sanger sequence data (Table 1); *TLR7* could not be genotyped due to high similarity between duplicate copies (see below). We observed 24 SNPs across all loci, of which 15 were non-synonymous (Table 1). Twenty-two SNPs were transitional and three were transversions (one SNP had three variants). The sequenced portion of *TLR3* did not contain any SNPs among the birds in our sample.

The majority of SNPs we observed were diallelic, the exception being one triallelic site in *TLR4*, segregating three alternative amino acids (Ile33, Leu33 or Phe33 – all non-polar). A non-conservative [61] amino-acid polymorphism, Asn (polar)/Asp (negatively charged), was the most common, occurring at four loci (*TLR4*, *TLR7*, *TLR15* and *TLR21*, Table 1). None of the SNPs we detected resulted in stop codons or frame-shift mutations.

All polymorphic TLRs except *TLR2A* contained at least one non-synonymous base substitution. For five of the genotyped loci (*TLR1LA*, *TLR1LB*, *TLR4*, *TLR5*, *TLR15*), all observed SNP haplotypes translated as different amino acid variants. For *TLR2B* and *TLR21*, multiple observed SNP haplotypes resulted in two and three amino-acid variants respectively. Although it is possible that synonymous substitutions have functional consequences [62], these are more difficult to predict than amino-acid variations.

After deducing the genotypes of unsampled founders from those of their offspring, we observed between two and five amino-acid variants per polymorphic locus among the Ulva Island founders (mean 2.7, Table 2). Examining the genotypes of founders only, observed heterozygosity of amino-acid variants at each TLR locus did not differ significantly from expectation under Hardy-Weinberg equilibrium (mean *H*_O_ = 0.417; *H*_E_ = 0.477, *p*-values for all comparisons >0.05, *N* = 12, Table 2). Individual founders were heterozygous at an average of 2.83 of seven polymorphic loci (SD = 1.4).

**Table 2:** Observed (*H*_O_) and expected heterozygosity (*H*_E_) estimates for seven Toll-like receptor loci genotyped among the 12 Ulva Island founders, based on amino-acid variants.

Locus^1^	Amino-acid variants	*H*_O_	*H*_E_	*p*-value^2^
*TLR1LA*	2	0.417	0.518	0.594
*TLR1LB*	2	0.250	0.518	0.104
*TLR2B*	2	0.500	0.591	0.143
*TLR4*	5	0.667	0.732	0.724
*TLR5*	3	0.500	0.489	1.000
*TLR15*	2	0.083	0.083	1.000
*TLR21*	3	0.500	0.409	1.000
Mean	2.7	0.417	0.477	

1 *TLR7* is excluded from this table because locus-specific heterozygosities could not be inferred (see Results).

2 Hardy-Weinberg exact test.

### Tests of selection

When comparing the distribution of synonymous and non-synonymous SNP variation within and between species (*P. a. rakiura* and *C. mexicanus*), we observed that the proportion of non-synonymous fixed differences between species for *TLR1LB* (51%) was approximately double the proportion of non-synonymous polymorphism within species (24%), a statistically significant result (McDonald-Kreitman test, *G* = 6.62, *p* = 0.010). The direction of the relationship at this locus indicates possible evidence of divergent positive selection at some time during the evolutionary history between the two species. Although there was no evidence of positive selection at the other loci we tested (*TLR1LA*, *TLR2A*, *TLR2B*, *TLR3*, *TLR4*, *TLR15* or *TLR21*), all loci except *TLR3* and *TLR21* showed the same pattern of excess fixed substitutions (contingency tables for all loci are provided in Table S1). For all tested loci, the number of fixed sites (both synonymous and non-synonymous) was greater than the number of variable sites, across both species, although it should be noted that the sample sizes for both species were small for most loci (see Methods).

### TLR7 duplication

Cloning results suggested a genomic duplication of *TLR7* in *P. a. rakiura*. All SNPs observed in Sanger sequence data were recovered in the cloning experiments; similarly, all variants found among clones were consistent with the Sanger sequencing data, with the exception of single base substitutions occurring in three reads (data not shown). Therefore, the error rate resulting from PCR, cloning and sequencing steps is low, as was expected given our use of high-fidelity polymerases (see Methods). Overall, we observed two main *TLR7* types: from individual “RU090” we observed one sequence each of type I and type II (*N* = 15 and 9 clones, respectively); from individual “RU152” we observed two sequences of type I (*N* = 15 and 2 clones) and four sequences of type II (*N* = 7, 5, 3 and 1 clones) (Table S2). This latter result could occur in two ways: either at least three gene-copies are present, or there are two gene-copies present as well as a high frequency of PCR chimera formation during pre-cloning amplification. Regions of high natural recombination are more likely to undergo chimera formation *in vitro*, as are highly similar sequences [63]. To examine whether this region of *TLR7* shows evidence of high natural recombination, we built a phylogenetic tree using available avian *TLR7* sequences, which revealed that duplicate copies of *TLR7* were more similar within species than between species (Figure S1). Although this tree structure may result from multiple recent gene duplication events, a simpler explanation is a single basal duplication event, with recent gene conversion. A high level of *in vitro* recombination is therefore possible here, which would imply two coamplifying copies of *TLR7*, with the additional haplotypes resulting from PCR artefacts. However, in one individual we observed five *TLR7* haplotypes that occurred in two or more independent PCR amplifications (Table S2), which is consistent with three coamplifying genomic copies of *TLR7*. Determining whether there are two or three copies of *TLR7* in *P. a. australis* would require extensive cloning of a larger number of individuals. The eight *TLR7* base positions that had previously appeared as “heterozygous” in Sanger sequencing data for all individuals are therefore interpreted as variant sites between *TLR7* paralogs – three sites are synonymous and four are non-synonymous (two sites are in the same codon). None of the observed *TLR7* clone haplotypes contained stop codons.

## Discussion

Variation in genes responsible for front-line immunity is likely an important target of natural selection, and can be used to obtain crucial insight into maintenance or loss of functional genetic diversity, and therefore adaptive potential, of threatened populations. Our study is the first to examine genetic diversity of Toll-like receptor genes in a bottlenecked population. Despite previous microsatellite evidence indicating reduced genetic variation in this population, we observed putatively functional variation in seven out of nine genotyped loci in this native New Zealand passerine. This variation in TLR loci highlights their promise as additional potential indicators of functional genetic diversity for the population and conservation genetics of bottlenecked species.

Of the polymorphic TLR loci we genotyped, levels of haplotype diversity were low (2–5 haplotypes per locus for seven loci) compared to levels reported for similarly-sized samples of *C. mexicanus* and lesser kestrel *Falco naumanni* (respectively 2–20 haplotypes for eight loci and 2–16 haplotypes for ten loci [23]). Both of these species, however, have much larger effective population sizes than *P. a. rakiura*. Low levels of genic diversity may be the result of purifying selection (e.g. [10]). It is interesting, however, that these genes displayed diversity at all, given the previous *P. a. rakiura* population bottleneck [42], [64], which is known to have been severe enough result in relatively low microsatellite diversity in this population (for example, only 53% of 70 species-specific microsatellite loci were variable in a sample of 12 Ulva Island birds, S. Townsend pers. comm. see also [44], [46]).

Despite observing a number of functional variants for most TLRs, the overall number of SNPs detected in this population was low (Table 1). TLR loci in other populations are typified by a high proportion of synonymous sequence polymorphism, as would be expected for a functional peptide with constraints on the majority of its sequence (e.g. fowl [40], pigs [65], [66], primates [67], rodents [10], [30]). The high number of monomorphic sites we observed in the current study is consistent with functional constraints over most of the sequence of each gene, but brings into focus the importance of the variation we did see: four loci showed only non-synonymous SNP variation (*TLR4*, *TLR5*, *TLR7* and *TLR15*, Table 1). This pattern may be evidence for a form of diversifying selection acting on TLR proteins in *P. a. rakiura*. While neutral diversity is more easily lost during the population bottleneck, certain amino-acid variants that may have functional consequences, either at present or in the past, may have been retained.

### Significance of variation in TLR genes in a threatened population

The overall pattern and context of our data suggests that variation at these loci has functional significance and, despite the limited sample size here, our analysis suggested that selection has been important in the evolution of at least one gene. For example, comparing *P. a. rakiura* sequence diversity to that of *C. mexicanus* indicated clear evidence of directional selection for *TLR1LB* since these two species diverged (McDonald-Kreitman test). In threatened populations, genetic diversity predictions for immune-related genes can be unclear, as an increase in the frequency of alleles with non-synonymous substitutions is predicted under heterozygote advantage, frequency dependent selection, or repeated rounds of directional selection (as would occur in a Red Queen-type scenario). Depending on the context, these processes may result in reduced genetic diversity after population bottlenecking [68].

Although our study does not allow us to predict specifically how the observed levels of diversity will relate to future evolutionary potential of the population, the presence of diversity *per se* will facilitate future studies into the effects of genetic drift and selection on these alleles. Innate immune responses are expected to play an important role in the evolution of resistance to novel diseases in wild populations, an expectation that was upheld in a recent study of the consequences of a *Mycoplasma* outbreak in wild house finches [69]. However, little is known about the role of TLR gene diversity in disease susceptibility of threatened species. Further empirical studies examining the evolutionary significance of TLR variation in threatened populations, and comparing the results to those obtained from MHC or neutral markers, alongside disease prevalence information, will be particularly informative.

### Ease of genotyping and genomic duplication

Most TLR loci were present in only single genomic copies, in contrast to avian studies of MHC which can find coamplification of many loci as well as genomic copy-number variation among individuals within a population (e.g. at least 20 MHC class II B loci were observed in common yellowthroat [37]). *TLR1* and *TLR2* are known to be duplicated in birds [21], and *TLR7* is probably duplicated in passerines [17], [23; current study]. *TLR2A* and *TLR2B* have conserved and divergent regions [17], [23], enabling design of locus-specific primers in the current study. These primers may be useful in other passerine species. *TLR7* duplicates were too similar in *P. a. rakiura* to design locus-specific primers, so unfortunately this gene could not be easily genotyped. Overall, while uncertainty in the number of co-amplifying genes and pseudogenes can be a major challenge of working with MHC in some taxa [6], resulting in a need for much greater genotyping effort, this problem is rare with TLR genes.

Our results were consistent with at least two genomic copies of *TLR7* in *P. a. rakiura*, similar to previous reports of *TLR7* duplication in *T. guttata* [17] and potentially *C. mexicanus* [23]. In *P. a. rakiura*, the two loci are clearly very similar, which implies that this is a recent independent duplication, or, if it is the same duplication as seen in other passerines, that gene conversion has prevented wholesale divergence. *TLR7* is thought to play a role in immunity to viruses (e.g. avian influenza in Pekin duck *Anas platyrhynchos domesticus* [70]) and loss or gain of TLR loci among derived lineages may reflect differences in selection pressures during the evolutionary histories of those taxa [14], [71]. The family of *P. a. rakiura*, Petroicidae, is a sister-group to the Passerida infraorder (which includes *T. guttata* and *C. mexicanus*) [72], indicating that this duplication may be basal to some passerine groups. As *TLR7* duplication has not been observed in any other vertebrate taxon to date [17], [23], it would be interesting to investigate *TLR7* in other passerines and closely-related avian orders to determine when duplication occurred relative to the passerine radiation.

### Conclusions

In summary, we found that the full family of TLRs could be successfully amplified in our bottlenecked population of *P. a. rakiura*, and that individual genotypes could be easily assigned for nine genes. Non-synonymous variation was present within the relatively small population of founder birds. The high proportion of non-synonymous variation for some genes suggests that these loci have been, and may still be, subject to natural selection, and therefore provide additional loci for addressing questions of functional genetic diversity in this and other threatened species. By presenting gene sequences, primers and diversity estimates for a bottlenecked population, we further enable study of TLRs in other conservation contexts, especially where the complexity of MHC may be prohibitive for economic and/or technical reasons.

## Supporting Information

Figure S1 **Neighbour-joining tree of** ***TLR7*** **sequences available for nine avian genera.**
(DOCX)

Table S1 **McDonald-Kreitman tests contrasting substitution and mutation rates for a comparison of** ***P. a. rakiura*** **TLR sequences with those of** ***C. mexicanus*** **** [**23**] **.**
(DOCX)

Table S2 **Alignment of clone haplotypes obtained for two individuals amplified at** ***TLR7*** **showing character states at variable sites; all other base positions were monomorphic.**
(DOCX)
