# Supplementary figures and images for: Variation at Innate Immunity Toll-Like Receptor Genes in a Bottlenecked Population of a New Zealand Robin
Source: PLoS One. 2012 Sep 14;7(9):e45011. doi: 10.1371/journal.pone.0045011 (PMC3443209; doi:10.1371/journal.pone.0045011)

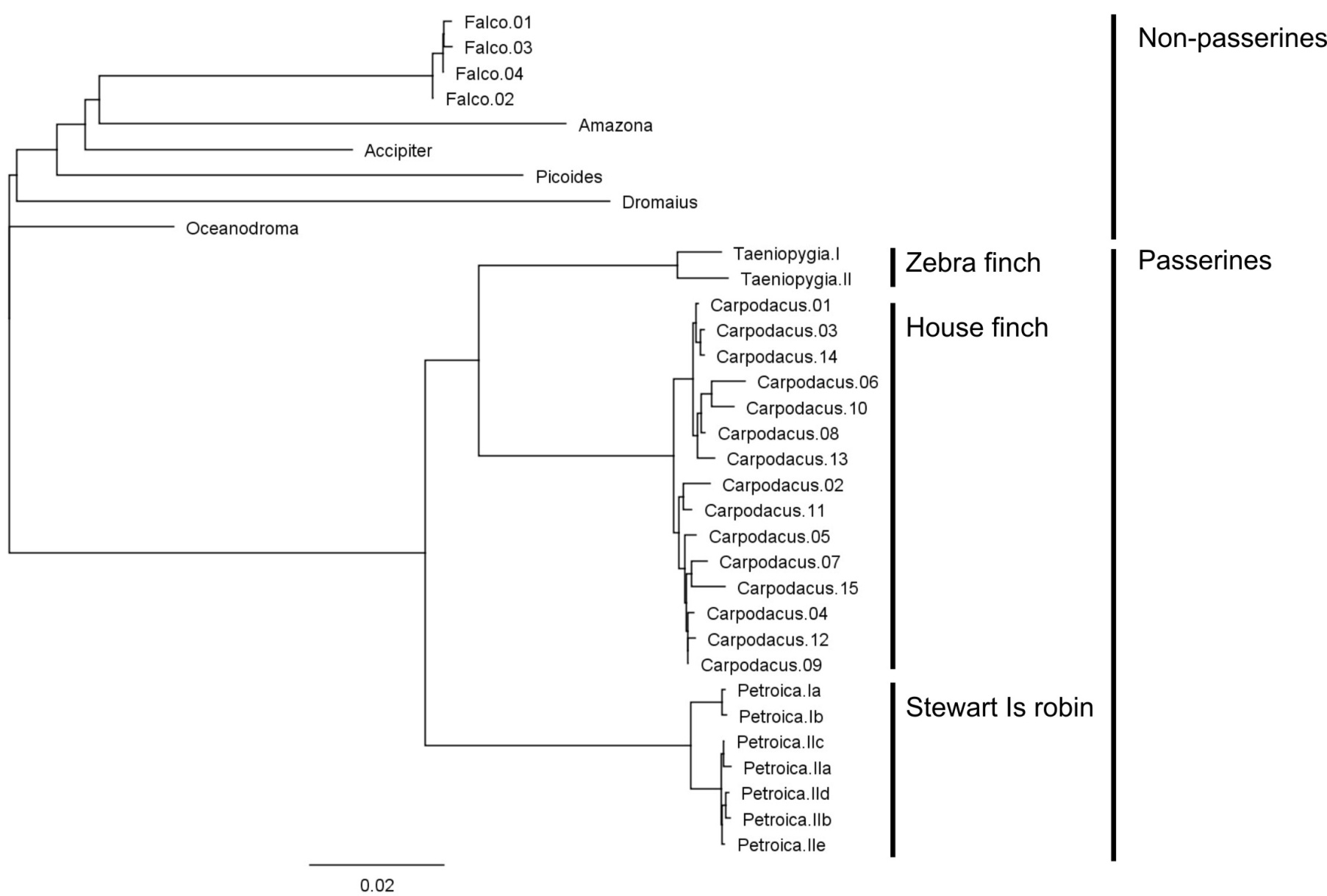


Figure S1: Neighbour-joining tree of *TLR7* sequences available for nine avian genera.

Supplement: Figure S1 — Neighbour-joining tree of TLR7 sequences available for nine avian genera. (DOCX) [file pone.0045011.s001.docx]
