# Supplementary material for: Variation at Innate Immunity Toll-Like Receptor Genes in a Bottlenecked Population of a New Zealand Robin
Source: PLoS One. 2012 Sep 14;7(9):e45011. doi: 10.1371/journal.pone.0045011 (PMC3443209; doi:10.1371/journal.pone.0045011)
Supplement: Table S1 — McDonald-Kreitman tests contrasting substitution and mutation rates for a comparison of P. a. rakiura TLR sequences with those of C. mexicanus [23] . (DOCX) [file pone.0045011.s002.docx]

Table S1: McDonald-Kreitman tests contrasting substitution and mutation rates for a comparison of *P. a. rakiura* TLR sequences with those of *C. mexicanus* [23].

| Locus | *C. mexicanus* sampled | *C. mexicanus* haplotypes | Aligned bases | Type of substitution | Fixed between species | Polymorphic within species | Total | *G*^1^ | *p* |
| --- | --- | --- | --- | --- | --- | --- | --- | --- | --- |
| *TLR1LA* | 51 | 62 | 1,152 | Synonymous | 50 | 27 | 77 | 0.394 | 0.530 |
|  |  |  |  | Non-synonymous | 42 | 18 | 60 |  |  |
|  |  |  |  | *Total* | *92* | *45* |  |  |  |
| *TLR1LB* | 13 | 20 | 944 | Synonymous | 49 | 22 | 71 | 6.62 | 0.010 |
|  |  |  |  | Non-synonymous | 50 | 7 | 57 |  |  |
|  |  |  |  | *Total* | *99* | *29* |  |  |  |
| *TLR2A* | 8 | 8 | 381 | Synonymous | 16 | 4 | 20 | 0.622 | 0.430 |
|  |  |  |  | Non-synonymous | 23 | 3 | 26 |  |  |
|  |  |  |  | *Total* | *39* | *7* |  |  |  |
| *TLR2B* | 8 | 5 | 315 | Synonymous | 13 | 3 | 16 | 0.007 | 0.935 |
|  |  |  |  | Non-synonymous | 14 | 3 | 17 |  |  |
|  |  |  |  | *Total* | *27* | *6* |  |  |  |
| *TLR3* | 8 | 9 | 952 | Synonymous | 31 | 5 | 36 | 2.04 | 0.153 |
|  |  |  |  | Non-synonymous | 14 | 6 | 20 |  |  |
|  |  |  |  | *Total* | *45* | *11* |  |  |  |
| *TLR4* | 8 | 14 | 649 | Synonymous | 20 | 8 | 28 | 0.289 | 0.591 |
|  |  |  |  | Non-synonymous | 40 | 12 | 52 |  |  |
|  |  |  |  | *Total* | *60* | *20* |  |  |  |
| *TLR15* | 8 | 16 | 1,279 | Synonymous | 43 | 18 | 61 | 0.810 | 0.368 |
|  |  |  |  | Non-synonymous | 61 | 18 | 79 |  |  |
|  |  |  |  | *Total* | *104* | *36* |  |  |  |
| *TLR21* | 4 | 2 | 618 | Synonymous | 25 | 2 | 27 | 0.594 | 0.441 |
|  |  |  |  | Non-synonymous | 18 | 3 | 21 |  |  |
|  |  |  |  | *Total* | *43* | *5* |  |  |  |

^1^ *G* = McDonald-Kreitman test statistic
