# Supplementary material for: Variation at Innate Immunity Toll-Like Receptor Genes in a Bottlenecked Population of a New Zealand Robin
Source: PLoS One. 2012 Sep 14;7(9):e45011. doi: 10.1371/journal.pone.0045011 (PMC3443209; doi:10.1371/journal.pone.0045011)
Supplement: Table S2 — Alignment of clone haplotypes obtained for two individuals amplified at TLR7 showing character states at variable sites; all other base positions were monomorphic. (DOCX) [file pone.0045011.s003.docx]

Table S2: Alignment of clone haplotypes obtained for two individuals amplified at *TLR7* showing character states at variable sites; all other base positions were monomorphic.

|  |  | Variable sites | | | Uniformly “heterozygous” sites | | | | | | | | Number of times observed^1^ | | | |  |
| --- | --- | --- | --- | --- | --- | --- | --- | --- | --- | --- | --- | --- | --- | --- | --- | --- | --- |
|  |  | 162 | 672 | 723 | 775 | 829 | 830 | 833 | 835 | 859 | 863 | 866 | AccuSure | Velocity | Phusion | Total | Genbank Accession |
| RU090 | Sanger data | A | Y | R | M | R | R | W | R | M | Y | M |  |  |  |  |  |
|  | Type I a | A | C | A | A | G | G | T | G | A | C | A | 9 | 4 | 2 | 15 |  |
|  | Type II a | A | T | G | C | A | A | A | A | C | T | C | 4 | 1 | 4 | 9 |  |
|  | Total |  |  |  |  |  |  |  |  |  |  |  | 13 | 5 | 6 | 24 |  |
| RU152 | Sanger data | R | C | R | M | R | R | W | R | M | Y | M |  |  |  |  |  |
|  | Type I a | A | C | A | A | G | G | T | G | A | C | A | 3 | 3 | 9 | 15 |  |
|  | Type I b | G | C | A | A | G | G | T | G | A | C | A |  | 1 | 1 | 2 |  |
|  | Type II b | G | C | A | C | A | A | A | A | C | T | C | 4 | 1 | 2 | 7 |  |
|  | Type II c | A | C | G | C | A | A | A | A | C | T | C | 3 |  | 2 | 5 |  |
|  | Type II d | A | C | A | C | A | A | A | A | C | T | C | 2 | 1 |  | 3 |  |
|  | Type II e | G | C | G | C | A | A | A | A | C | T | C | 1 |  |  | 1 |  |
|  | Total |  |  |  |  |  |  |  |  |  |  |  | 13 | 6 | 14 | 33 |  |

^1^ Original PCR product amplified using three alternative proofreading enzymes
